# Phosphotyrosine profiling of curcumin-induced signaling

**DOI:** 10.1186/s12014-016-9114-0

**Published:** 2016-06-15

**Authors:** Gajanan Sathe, Sneha M. Pinto, Nazia Syed, Vishalakshi Nanjappa, Hitendra S. Solanki, Santosh Renuse, Sandip Chavan, Aafaque Ahmad Khan, Arun H. Patil, Raja Sekhar Nirujogi, Bipin Nair, Premendu Prakash Mathur, T. S. Keshava Prasad, Harsha Gowda, Aditi Chatterjee

**Affiliations:** Institute of Bioinformatics, Unit I, 7th Floor, Discoverer Building, International Tech Park, Bangalore, 560066 India; Manipal University, Madhav Nagar, Manipal, 576104 India; YU-IOB Center for Systems Biology and Molecular Medicine, Yenepoya University, Mangalore, 575018 India; Department of Biochemistry and Molecular Biology, Pondicherry University, Puducherry, 605014 India; Amrita School of Biotechnology, Amrita University, Kollam, 690525 India; School of Biotechnology, KIIT University, Bhubaneswar, 751024 India; Centre of Excellence in Bioinformatics, School of Life Sciences, Pondicherry University, Puducherry, 605014 India; NIMHANS-IOB Proteomics and Bioinformatics Laboratory, Neurobiology Research Centre, National Institute of Mental Health and Neurosciences, Bangalore, 560029 India

**Keywords:** Oral cancer, Phosphoproteomics, In vivo labeling, Curcumin

## Abstract

**Background:**

Curcumin, derived from the rhizome *Curcuma longa*, is a natural anti-cancer agent and has been shown to inhibit proliferation and survival of tumor cells. Although the anti-cancer effects of curcumin are well established, detailed understanding of the signaling pathways altered by curcumin is still lacking. In this study, we carried out SILAC-based quantitative proteomic analysis of a HNSCC cell line (CAL 27) to investigate tyrosine signaling in response to curcumin.

**Results:**

Using high resolution Orbitrap Fusion Tribrid Fourier transform mass spectrometer, we identified 627 phosphotyrosine sites mapping to 359 proteins. We observed alterations in the level of phosphorylation of 304 sites corresponding to 197 proteins upon curcumin treatment. We report here for the first time, curcumin-induced alterations in the phosphorylation of several kinases including TNK2, FRK, AXL, MAPK12 and phosphatases such as PTPN6, PTPRK, and INPPL1 among others. Pathway analysis revealed that the proteins differentially phosphorylated in response to curcumin are known to be involved in focal adhesion kinase signaling and actin cytoskeleton reorganization.

**Conclusions:**

The study indicates that curcumin may regulate cellular processes such as proliferation and migration through perturbation of the focal adhesion kinase pathway. This is the first quantitative phosphoproteomics-based study demonstrating the signaling events that are altered in response to curcumin. Considering the importance of curcumin as an anti-cancer agent, this study will significantly improve the current knowledge of curcumin-mediated signaling in cancer.

**Electronic supplementary material:**

The online version of this article (doi:10.1186/s12014-016-9114-0) contains supplementary material, which is available to authorized users.

## Background

Curcumin, a bioactive compound derived from the rhizome *Curcuma longa* has been known for its chemopreventive and chemotherapeutic potential [1, 2]. It is a polyphenol compound with an aromatic ring structure connected by two α, β-unsaturated carbonyl groups and has been extensively studied for its diverse range of biological activities, including anti-inflammatory, anti-oxidant, analgesic and antiseptic properties [3–6]. It has attracted widespread attention as a potential therapeutic agent because of its pharmacological effects. The anti-tumor activity of curcumin is thought to be mediated through multiple mechanisms. At the molecular level, curcumin is known to induce apoptosis in a wide array of cancer cells including human colon, stomach, liver, breast, and prostate cancers [7–11]. It is known to mediate its effects by inhibition of anti-apoptotic markers such as Bcl-2, Bcl-xL, Survivin, and increased expression of pro-apoptotic factors such as Bax, Bad, Bak, PUMA, Bim, Noxa and TRAIL-R1 [12–14]. Curcumin has also been shown to inhibit cellular proliferation by downregulating several oncogenes such as EGFR, HER-2, PI3K/AKT, MAPK and upregulating the expression of various tumor suppressor genes such as p21WAF1/CIP1, p27KIP1 and p53 [15–19]. Furthermore, in vivo studies using animal models of skin and oral cancer have shown that curcumin inhibits tumor initiation and progression [20, 21].

Curcumin mediates it effect by targeting multiple cell growth signaling pathways, including PI3K-AKT, mTOR, EGFR and TGF-β signaling, amongst others [22–25]. It has been reported to cause a dose and time-dependent decrease in the phosphorylation of AKT and mTOR leading to decreased cellular proliferation and survival [26]. Curcumin has also been reported to induce the suppression of NF-κΒ and IΚΚ activation in melanoma cells and inhibit JNK signaling and STAT3 activation which in turn decreases the expression of pro-survival proteins [27–29]. Currently, information pertaining to curcumin-mediated tyrosine phosphoproteome signaling is minimal and the detailed signaling mechanism responsible for various biological effects of curcumin remains elusive. Understanding the signaling pathways responsible for its anti-neoplastic activity will provide avenues to identify novel therapeutic targets for cancers.

Aberrant activation of signaling pathways mediated by kinases is a common phenomenon in multiple malignancies. Tyrosine kinases regulate various cellular processes such as cell proliferation, differentiation, motility, cell cycle homeostasis, transcriptional regulation, and metabolism through reversible phosphorylation [30]. Although several studies have been carried out to characterize curcumin-induced alterations in cellular proteome of neuroblastoma [31], breast [32], gastric [11] and cervical cancers [33]; no effort have been made to study the changes in tyrosine signaling mediated by curcumin using quantitative phosphoproteomics approach.

In this study, we carried out SILAC-based quantitative proteomic analysis of CAL 27 cells (a HNSCC cell line) to investigate the tyrosine signaling in response to curcumin. Previous studies have reported curcumin-induced apoptosis and decreased cell proliferation in CAL 27 [34, 35]. Combining SILAC with anti-phosphotyrosine antibody-based enrichment and high resolution mass spectrometry analysis enabled identification of 627 unique phosphorylation sites mapping to 359 proteins including several novel curcumin-regulated phosphorylation events. Further, bioinformatics analysis identified perturbations in pathways regulating focal adhesions and actin cytoskeleton in curcumin-treated cells suggesting that curcumin may mediate its anti-proliferative effects through these pathways.

## Methods

### Reagents

Anti-phosphotyrosine rabbit monoclonal antibody (P-Tyr-1000) beads, MAPK, EPHA2 antibody were obtained from Cell Signaling Technology (Danvers, MA) and 4G10 anti-phosphotyrosine (HRP conjugated) antibody was purchased from Millipore (Billerica, MA). Curcumin was purchased from Sigma (St. Louis, MO). TPCK-treated trypsin was from Worthington Biochemical Corp. (Lakewood, NJ). DMEM with and without lysine and arginine, fetal bovine serum (FBS), l-glutamine, and antibiotics were purchased from Invitrogen (Carlsbad, CA). SILAC amino acids, ^13^C_6_-Lysine and ^13^C_6_-Arginine, were obtained from Cambridge Isotope Laboratories (Andover, MA). All other reagents used in this study were from Fisher Scientific (Pittsburgh, PA).

### MTT cell proliferation assay

To determine the effect of curcumin on CAL 27 cells, MTT (3-(4,5-dimethylthiazolyl-2)-2,5-diphenyltetrazolium bromide) assay was carried out according to manufacturer’s protocol (ATCC 30-1010K). Briefly, cells were seeded at a density of 8 × 10^3^ and treated with curcumin at varying concentration (0–25 µM) for 48 h. After incubation, MTT reagent was added and incubated for 2–4 h until the purple precipitate was formed. Purple crystals were solubilised using 100 µl of detergent solution and left at room temperature for 2 h. Further, the absorbance was read at 570 and 650 nm.

### Cell culture and SILAC labeling

CAL 27 cells was obtained from American Type Culture Collection (ATCC, Manassas, VA). CAL 27 cells were maintained in a humidified incubator at 37 °C with 5 % CO_2_. The cells were cultured in DMEM containing heavy stable isotopic forms of lysine and arginine (^13^C_6_l-lysine and ^13^C_6_l-arginine), 10 % FBS and 1 % penicillin/streptomycin mixture (SILAC media). CAL 27 cells were also grown in regular DMEM containing 10 % FBS and 1 % penicillin/streptomycin mixture. When cells reached 70 % confluence, the cells were subjected to serum starvation for 8 h. Post-serum starvation, cells cultured in SILAC media were treated with DMSO and cells cultured in regular DMEM were treated with curcumin (11.5 µm) for 4 h. Following 4 h treatment, the cells from both conditions were washed with ice cold 1X phosphate buffer saline (PBS) thrice and harvested in lysis buffer.

### Cell lysis and protein digestion

The DMSO (vehicle control) and curcumin treated CAL 27 cells were lysed in lysis buffer (20 mM HEPES pH 8.0, 9 M urea, 1 mM sodium orthovanadate, 2.5 mM sodium pyrophosphate, 1 mM β-glycerophosphate), sonicated and centrifuged at 16,000×*g* for 20 min. Protein concentration was determined using BCA assay (Pierce, Waltham, MA). Equal amounts of protein (20 mg) were mixed and the cysteine residues were reduced and alkylated with 5 mM DTT for 20 min at 60 °C and 10 mM iodoacetamide for 10 min at room temperature respectively. For trypsin digestion, the samples were diluted such that urea was <2 M with 20 mM HEPES, pH 8.0 and subjected to digestion with TPCK treated trypsin (Worthington Biochemical Corp, Lakewood, NJ) for 12–16 h at room temperature. Protein digests were acidified by 1 % trifluoroacetic acid (TFA) and desalted using C_18_ Sep-Pak cartridge (Waters, Cat#WAT051910) and lyophilized.

### Immunoaffinity purification of tyrosine phosphopeptides

The lyophilized peptide mixtures were dissolved in in IAP buffer containing 50 mM MOPS pH 7.2, 10 mM sodium phosphate and 50 mM NaCl. Prior to phospho-tyrosine enrichment, the P-Tyr-1000 beads (Cell Signaling Technology, Danvers, MA) were washed twice with IAP buffer at 4 °C. The peptide mixture was then incubated with P-Tyr-1000 beads for 30 min with gentle rotation. To remove non-specifically bound peptides, the beads were washed thrice with ice cold IAP buffer and twice with ice cold water. Elution of enriched peptides from beads was carried out at room temperature using 0.15 % TFA. This step was repeated twice. This was followed by clean up of the samples using C_18_ StageTips as described earlier [36].

### LC-MS/MS analysis of enriched peptides

The enriched phosphotyrosine containing peptides were analyzed on Orbitrap Fusion Tribrid mass spectrometer (Thermo Electron, Bremen, Germany) interfaced with Easy-nLC II nanoflow liquid chromatography system (Thermo Scientific, Odense, Denmark). Peptide digests were reconstituted in 0.1 % formic acid and loaded onto trap column packed (75 µm × 2 cm) with Magic C18 AQ (Michrom Bioresources, Inc., Auburn, CA) at a flow rate of 3µL/min. Peptides were separated on an analytical column (75 µm × 20 cm) at a flow rate of 400 nL/min using a step gradient of 5–25 % solvent B (0.1 % formic acid in 95 % acetonitrile) for first 110 min and 25–40 % solvent B for 110–140 min. The total run time was set to 180 min. Mass spectrometer was operated in data-dependent acquisition mode. A survey full scan MS (from m/z 350–1700) was acquired in the Orbitrap with resolution of 120,000 at 400 m/z. Most intense fifteen precursor ions with charge state ≥2 were isolated and fragmented using HCD fragmentation with 30 % normalized collision energy and detected at a mass resolution of 30,000 at 400 m/z. Dynamic exclusion was set for 30 s with a 10 ppm mass window.

### Data analysis

The MS/MS searches were carried out using MASCOT (Version 2.2.0) and SEQUEST search algorithms against RefSeq human protein database (version 65 containing 34,453 entries with common contaminants) using Proteome Discoverer 1.4 (Version 1.4.0.288 Thermo Fisher Scientific, Bremen, Germany). The workflow for both algorithms included spectrum selector, MASCOT, SEQUEST search nodes, peptide validator, event detector, precursor quantifier, and phosphoRS nodes. Oxidation of methionine, phosphorylation at serine, threonine and tyrosine (+79.966 Da) and SILAC labeling (^13^C_6_) at lysine and arginine (+6.02013 Da) were set as variable modifications and carbamidomethylation of cysteine was set as a fixed modification. MS and MS/MS mass tolerances were set to 5 ppm and 0.025 Da, respectively. Trypsin was specified as protease and a maximum of one missed cleavage was allowed. Target-decoy database searches used for calculation of false discovery rate (FDR) and for peptide identification FDR was set at 1 %. Quantitation node was used for calculation of SILAC ratio for each phosphopeptide-spectrum match (phosphoPSM) and probability of the phosphorylation site was calculated using phosphoRS 3.1 node in the Proteome Discoverer. The SILAC ratios were normalized based protein median. Phosphopeptides with >75 % localization probability were considered for further analysis [37].

### Availability of data

The mass spectrometry derived data have been deposited to the ProteomeXchange Consortium (http://proteomecentral.proteomexchange.org) via the PRIDE partner repository with the dataset identifier PXD002097.

### Bioinformatics analysis

Molecular function of phosphoproteins was obtained from Human Protein Reference Database (HPRD) (http://www.hprd.org/) [38]. The differentially phosphorylated proteins upon curcumin treatment were mapped to gene networks available in Ingenuity Systems Pathway Analysis (IPA) platform (https://analysis.ingenuity.com) and ranked based on the score. Network analysis was performed with the significantly enriched genes. KEGG pathway mapping of curcumin-regulated phosphoproteins was performed using the DAVID bioinformatics functional annotation tool [39]. Identification of enriched motifs was carried out using motif-X algorithm [40]. A 15 amino acids phospho window was used for extracting consensus motif. The significance threshold was set to *p* < 0.02 and the minimum occurrence of motifs was set to 10.

### Western blot analysis

CAL 27 cells were cultured in DMEM in 37 °C humidified 5 % CO_2_ incubator. The cells were serum starved for 8 h prior to curcumin treatment. The cells were treated with either vehicle control (DMSO) or curcumin for 1 and 4 h. Cells were lysed in modified RIPA buffer (50 mM Tris-HCl, pH 7.4, 150 mM NaCl, 1 mM EDTA, 1 % Nonidet P-40, 0.25 % sodium deoxycholate, and 1 mM sodium orthovanadate in the presence of protease inhibitors) followed by centrifugation. The protein lysates were resolved using SDS-PAGE and Western blot analysis was performed using phospho-tyrosine antibody 4G10. Western blot analysis were performed on CAL 27 lysate (untreated and curcumin treated) using anti-phospho and total MAPK1/MAPK3 and EPHA2 antibodies. β-actin was used as a loading control.

### Colony formation and invasion assays

Colony formation and invasion assays were performed as described previously [41]. Briefly, CAL 27 cells were seeded at a density of 3 × 10^3^ cells into a 6-well plate with complete media. After 24 h, cells were treated with curcumin and cells treated with DMSO served as control. Cell colonies were allowed to grow for 10 days. Then, the colonies were fixed using methanol and stained with 4 % methylene blue. The number of colonies per well were counted. All experiments were done in duplicate. All experiments were repeated thrice.

The effect of curcumin on the invasion potential of CAL 27 cells was assessed in vitro in a transwell system (BD Biosciences, San Jose, CA) using Matrigel coated filters as described previously [42]. DMSO and curcumin treated CAL 27 cells at a density of 2 × 10^4^ were suspended in 500 µl of serum free media and seeded on the Matrigel-coated PET membrane in the upper compartment. The lower compartment was filled with complete growth media and the plates were incubated at 37 °C for 48 h. At the end of the incubation, the upper surface of the membrane was wiped with a cotton-tip applicator to remove non-migratory cells. Cells that migrated to bottom side of membrane were fixed and stained using 4 % methylene blue. Each measurement was performed in duplicate. All experiments were repeated thrice.

## Results and discussion

### Curcumin inhibits cellular proliferation, invasion and colony forming ability of CAL 27 cells

**Fig. 1:**
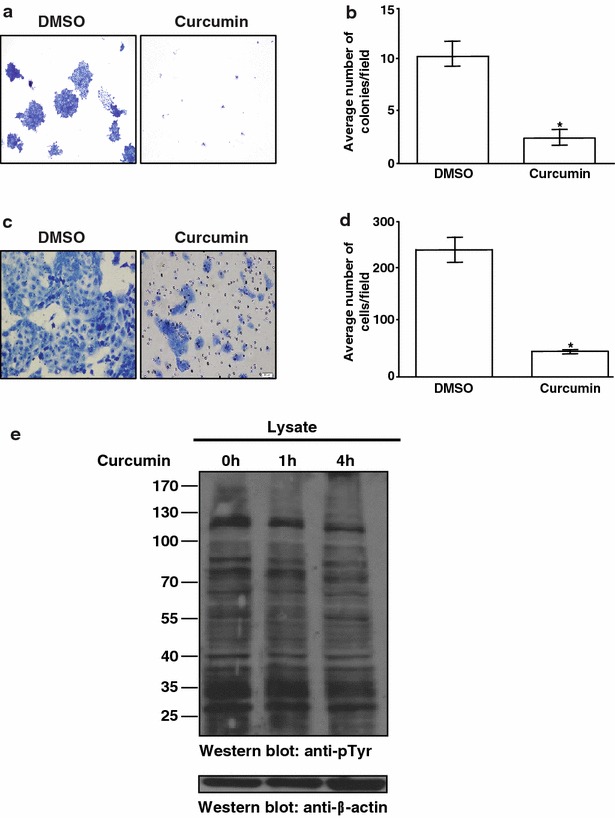
Curcumin treatment leads to decrease in invasive property and colony formation ability. **a** Colony formation assay following treatment of CAL 27 cells with curcumin or vehicle (DMSO). **b** A graphical representation of the colony forming ability of the CAL 27 cells upon treatment with curcumin or vehicle (DMSO). **c** CAL 27 cells were treated with either curcumin or vehicle (DMSO) for 48 h and invasive property of the cells were monitored. Cells that migrated are visualized using methylene blue. **d** A graphical representation of the invasive ability of the CAL 27 cells in presence of curcumin or vehicle (DMSO). **e** Phosphotyrosine profile of CAL 27 cells treated with curcumin for 0, 1 and 4 h analyzed by Western blotting using anti-phosphotyrosine antibody (Cat # 16-316)

To determine the effect of curcumin on cell survival, CAL 27 cells were treated with curcumin at varying concentrations (0–25 µM) and subjected to MTT assay. CAL 27 cells showed a decrease in cell viability in presence of curcumin (Additional file 1: Fig.S1A). Next, we studied the colony forming ability of CAL 27 cells in presence of curcumin. There was a significant reduction in the colony forming ability of the cells in the presence of curcumin compared to control cells (Fig. 1a, b) (p value <0.005). We also observed a significant decrease in the invasive ability of CAL 27 cells upon curcumin treatment (Fig. 1c, d) (p value <0.001). Taken together, these results suggest that curcumin inhibits cellular proliferation and metastatic potential of CAL 27 cells.

### Quantitative phosphoproteomic analysis of curcumin induced signaling

**Fig. 2:**
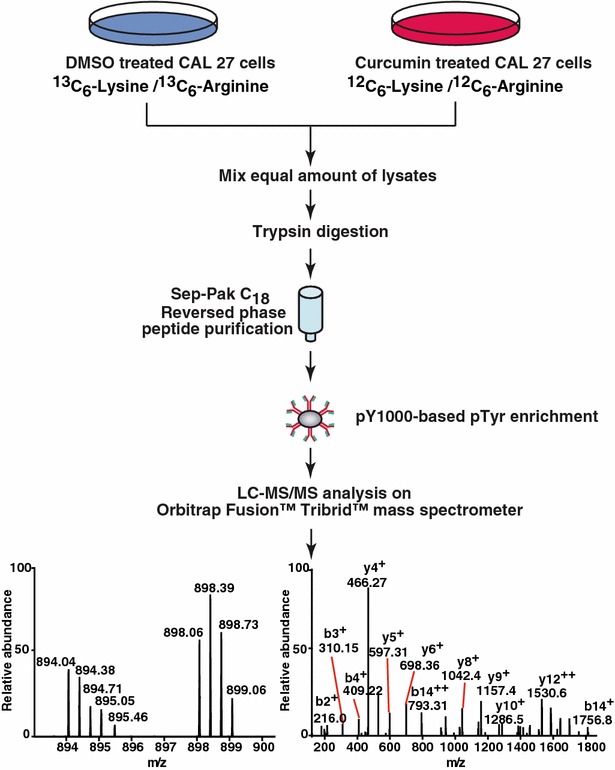
Workflow employed to identify changes in the phosphorylation status in response to curcumin. CAL 27 cells were cultured in “light” or “heavy” SILAC medium. The cells grown in ‘light’ medium were treated with curcumin for 4 h and the cells grown in ‘’heavy medium’’ were treated with vehicle (DMSO). The samples were subjected to trypsin digestion and enriched for phosphopeptides using anti-phosphotyrosine antibodies for enrichment of tyrosine-phosphorylated peptides. The enriched phosphopeptides were analyzed by LC-MS/MS

In order to characterize signaling mechanism through which curcumin functions, CAL 27 cells were treated with curcumin for different durations to identify the time point when tyrosine signaling is affected. An initial immunoblot analysis of tyrosine phosphorylation status in CAL 27 cells upon curcumin exposure for 0, 1 and 4 h indicated a moderate decrease in tyrosine signaling at 4 h of curcumin treatment (Fig. 1e). We investigated the molecular mechanism of curcumin-induced signaling using SILAC-based quantitative phosphoproteomic analysis. Cells treated with DMSO (control cells) were adapted to ‘heavy’ SILAC media whereas the cells grown in ‘regular’ media were treated with curcumin for 4 h. Post curcumin treatment, the cells were lysed, equal amounts of lysates were pooled and digested with trypsin. The phosphotyrosine peptides were enriched by immunoaffinity purification and analyzed on Orbitrap Fusion Tribrid mass spectrometer. The schematic workflow of SILAC-based phosphoproteomics analysis is shown in Fig. 2.

**Fig. 3:**
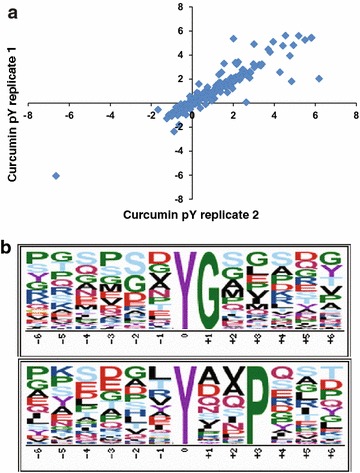
Summary statistics of the analysis. **a** Correlation of the normalized log2 SILAC ratio between triplicate measurements of anti-phosphotyrosine antibody enrichment method (Pearson correlation coefficient 0.8). **b** Curcumin-induced differentially regulated motifs. The motifs that were identified to be enriched in curcumin-induced differentially regulated phosphorylation sites dataset are depicted

LC-MS/MS analysis of phosphotyrosine enriched sample was carried out in triplicate and the acquired mass spectrometry data was processed and searched using MASCOT and SEQUEST search algorithms. We identified 5368 phosphopeptide-spectral matches (Additional file 2: Table S1) with a false discovery rate (FDR) of 1 %. Data acquired in triplicate showed good correlation (Pearson correlation coefficient 0.8) (Fig. 3a). PhosphoRS probability cutoff of 75 % was used for unambiguous localization of phosphorylation sites which lead to the identification of 672 unique phosphopeptides corresponding to 627 phosphorylation sites mapping to 359 proteins (Additional file 3: Table S2). Using 1.5-fold cutoff for hyperphosphorylation and 0.67-fold cutoff for decreased phosphorylation (hypophosphorylation) events, we identified 265 hyperphosphorylated and 40 hypophosphorylated phosphopeptides upon curcumin treatment. These curcumin-regulated phosphopeptides correspond to 187 proteins.

### Functional analysis of curcumin regulated phosphoproteome

Since we observed widespread signaling alterations upon curcumin treatment, we next performed bioinformatics analysis of the differentially phosphorylated proteins to categorize them based on their cellular localization and biological function. The classifications were based on annotations in HPRD, a Gene Ontology (GO) compliant database [38, 43]. Our analysis revealed that majority of the curcumin regulated proteins were localized in the cytoplasm (43 %) followed by plasma membrane (24 %), nucleus (19 %) and cytoskeleton (5 %) (Additional file 1: Fig. S1B). The proteins which were found to be differentially expressed upon curcumin exposure were involved in a broad range of molecular functions. Majority of the proteins were found to be involved in catalytic activity, binding activity, and enzyme regulatory activity (Additional file 1: Fig. S1C). A major category (16 %) of proteins regulated by curcumin were adaptor proteins. These include GRB2-associated-binding protein (GAB1), GRB2-associated-binding protein 2 (GAB2), SH2 domain-containing adapter protein B (SHB) and phosphoprotein membrane anchor with glycosphingolipid microdomains 1 (PAG1) that were differentially phosphorylated upon curcumin treatment. The other major categories of proteins identified were kinases (15 %), cytoskeletal proteins (14 %), membrane and cell junction proteins (13 %) and the transport/carrier proteins. Classification based on the biological process revealed that majority of the proteins regulated by curcumin were involved in cell communication (44 %), followed by cell growth (18 %) and metabolic processes (12 %). (Additional file 1: Fig. S1D). We also employed Ingenuity Pathway analysis (IPA) analysis tool to group differentially phosphorylated proteins into networks and canonical pathways to determine altered cellular activities upon curcumin treatment. The top networks identified through this analysis included cellular movement, cancer and cellular development, cell-to-cell signaling and interaction. The proteins identified in the most significant biological network correlate with FAK and PI3K signaling pathways (Table 1).

**Table 1:** The top five biological networks identified by IPA

Top functions (Networks)	Associated molecules	Score	Focus molecule
Cellular movement, cell morphology, cellular function and maintenance	Catenin, *BAG3, BCAR1*, BCR, c-Src, *CAV1, CRK, CTNND1, DOCK1, EPHA2*, F Actin, *FAK*,*GAB1*, Igm, *LASP1, MLLT4, MYO1E*, NFkB, p85, *PDLIM1, PEAK1*, PI3K p85, PLC gamma, *PLCG1, PPP1CA, PPP1CB, PTPN6, PTPN11*, Ras, Rock, *SHC1, SLC9A1, TJP1, TJP2, TNK2*	37	23
Cellular movement, cancer, cellular development	*ADAM9, ANXA2*, Ap1, *AXL, BCAR3*, calpain, *CDH1, COL17A1, CTTN, EGFR, EPS15, ERBB2,* ERK1/2, *FAM120A*, G protein alphi, *INPPL1*, *IQGAP1, ITGB4, JAK2,* Mapk, Mek, Mmp, *PIK3R1, PTK2, PTPRE, PTPRK*, Rac, Rap1, *RIN1, SCAMP3, SDCBP*, Shc, SRC,Vegf	35	22
Cardiac arrythmia, cardiovascular disease, organismal injury and abnormalities	*ARHGAP35*, Cg, *CLDN1*, *DSG2, DSP,* ERK, ER, *FLNB*, FSH, *FUBP3*, Growth harmone, HDAC, *HNRNPA1, HNRNPA2B1, HNRNPK*, Hsp70, Hsp90, *HSP90AA1*, JNK, *JUP, LDLR*, LH, *PCBP2, PCDH7*, *PKP2, PLIN3*, Ras homologue, *RASAL2,* RNA polymerase II, Secretase gamma, *SPTAN1, TNS3, TUBA1A, TUBA1C*	33	21
Cellular assembly and organization, cellular function and maintenance, cell-to-cell signaling and interaction	*ABLIM1*, Akt, *ANXA1, CCDC88A*, CD3, *CFL1, CRKL, ERRFI1*, Fibrinogen, G protein beta gamma, Gsk3, Hsp27, IgG, LDL, MAP2K1/2, *MAPK1, MAPK3, MAPK13, MAPK14, NEDD9*, P38 MAPK, *PAG1*, PDGF BB, PI3 K (complex), PI3 K (family), pkc, PP2A, *PRKCD, PTK2B, SHANK2*, STAT5a/b, *SYK*, TCR, *VAV2, YES1*	26	18
Embryonic development, hair and skin development and function, organ development	*AHNAK*, AIM1, CASP14, COL7A1, CTNNB1, *EPHA1, EPHB2, ERBB2, ERBB2IP*, ETV5, FOXQ1, GCHFR, GRB2, HENMT1, *HNRNPA3, KRT5*, KRT7, *KRT14*, KRT16, KRT80, NRCAM, OCIAD2, PABPC4, PHLDB1, *PKP3, PKP4*, QPCT, RPS17, SCG5, SCGB2A2, *SHB*, SMARCA4, *STAT5A*, TSPAN13, *ZNF185*	17	13

Motif analysis was carried out to find over-representation of motifs among differentially phosphorylated sequence up on curcumin treatment. Motif analysis using motif-X algorithm enabled identification of two distinct phosphorylation motifs “pYxxP” and “pYG” (Fig. 3b). pYxxP is a proline directed motif where tyrosine is followed by a proline at +3 position and is known to be the recognition motif for phosphorylation by ABL kinase (ABL1). ABL kinase plays an important role in the regulation of cell proliferation, migration and cell survival [44]. pYG is a glycine directed motif where tyrosine is followed by glycine at +1 position and is a known motif for phosphorylation by Src and Lck kinases. Src family kinases (SFKs) are known to be involved in cell growth, division, migration, and survival signaling pathways [45].

### Protein kinases and phosphatases altered in curcumin-mediated signaling

 Protein kinases and phosphatases play a critical role in the regulation of signaling networks. In this study, we identified 205 proteins which were differentially phosphorylated upon curcumin treatment. This involved 29 protein kinases including TNK2, FRK and AXL and 6 protein phosphatases such as PTPN6, PTPN11, PTPRK and INPPL1. Of these, the role of 18 protein kinases and 5 protein phosphatases have not been reported previously in curcumin-mediated signaling. A partial list of protein kinases and phosphatases altered on curcumin treatment has been listed in Table 2.

**Table 2:** A partial list of novel kinases/phosphatases regulated by curcumin

Gene symbol	Protein	Site	Phosphopeptide sequence	Fold change (curcumin/DMSO)
*PTPN6*	Tyrosine-protein phosphatase non-receptor type 6	Y536	GQESEyGNITYPPAMK	5.1
*ABL2*	Abelson tyrosine-protein kinase 2	Y647	yELTGLPEQDR	4.4
*INPPL1*	Phosphatidylinositol 3,4,5-trisphosphate 5-phosphatase 2	Y986	NSFNNPAyYVLEGVPHQLLPPEPPSPAR	2.8
*MAPK11*	Mitogen-activated protein kinase 11	Y182	QADEEMTGyVATR	2.2
*PTPRK*	Receptor-type tyrosine-protein phosphatase kappa	Y858	CEGTESPyQTGQLHPAIR	2.5
*FRK*	Fyn-related Src family tyrosine kinase	Y497	LEDYFETDSSySDANNFIR	0.5
*PEAK1*	Pseudopodium enriched atypical kinase 1	Y635	IVINPNAyDNLAIYK	0.5
*TNK2*	Activated CDC42 kinase 1	Y827	yATPQVIQAPGPR	0.5
*AXL*	Tyrosine-protein kinase receptor UFO	Y598	yVLCPSTTPSPAQPADR	0.5
*PTPRE*	Receptor-type tyrosine-protein phosphatase epsilon	Y638	VVQDFIDIFSDyANFK	0.5

**Fig. 4:**
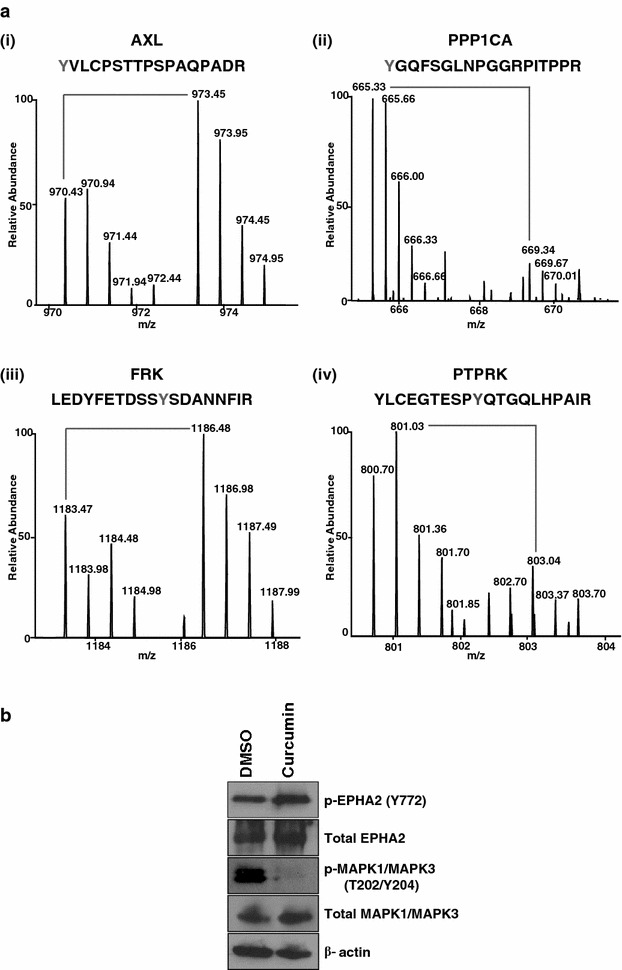
Curcumin regulated phosphoproteome. **a** Representative MS spectra of phosphorylated kinases/phosphatases. **a** *i*, *iii*, phosphorylation of peptides on kinases (AXL and FRK); **a** *ii*, *iv* phosphatases (PPP1CA and PTPRK) was differentially phosphorylated as evidenced by MS spectra showing the changes in the relative abundance of phosphopeptides. **b** Proteins identified to be differentially phosphorylated upon curcumin treatment from our mass spectrometry data were validated by Western blot using anti-phospho antibodies for pEPHA2 (Y772) and pMAPK1/MAPK3 (T202/Y204). Total expression was probed using anti-EPHA2 and anti-MAPK1/MAPK3

Amongst the protein kinases regulated by curcumin, we identified receptor tyrosine kinases including AXL, ERBB2 and EPHA1. Overexpression of AXL has been reported in various cancers such as thyroid carcinoma [46], renal cell carcinoma [47] and esophageal adenocarcinoma [48]. It is reported to be a potential biomarker for both early diagnosis and prognosis of oral squamous cell carcinoma [49]. Expression of AXL inversely correlates with survival of breast cancer patients with primary tumor and metastasis [50]. Our data indicates decrease in phosphorylation of AXL at Y598 upon curcumin treatment [Fig. 4a (i)]. Y598 is located in the tyrosine kinase domain of the AXL and can play an important role in the activation of tyrosine kinase activity of AXL to angiogenic responses and tumor neovascularization [51]. We also identified several members of MAPK signaling pathway to be differentially phosphorylated upon curcumin treatment. MAPK signaling has been shown to regulate cellular proliferation, particularly via the ERK-1/2 pathway [52]. This signaling pathway plays an important role in the growth, development, proliferation, and malignant transformation of cells. ERK-1/2 pathway is aberrantly activated in a variety of tumors and facilitates invasion and metastasis through activation of downstream effectors [53]. Curcumin is known to inhibit phosphorylation of ERK1/2 in CNE-2Z cells in a dose dependent manner [54]. We observed a decreased phosphorylation of the conserved residues T202/Y204 of ERK1/2 (MAPK3) upon curcumin treatment which is corroborated by western blot analysis as well (Fig. 4b).

In addition to kinases, we also observed alterations in the activity of multiple phosphatases upon curcumin treatment. Hyperphosphorylation of several phosphatases including, protein phosphatase 1 (PPP1CB), protein tyrosine phosphatase, non-receptor type 6 (PTPN6) and protein tyrosine, non-receptor type 11 (PTPN11) was observed upon curcumin treatment. PPP1CB and PPP1CA [Fig. 4a (ii)] are catalytic subunits of protein phosphatase 1 PP1, a serine/threonine specific protein phosphatase involved in the regulation of cell division and glycogen metabolism. PPP1CA is known to cause cell cycle arrest thereby preventing oncogenic transformation [55]. PTPN6 and PTPN11 are members of the protein tyrosine phosphatase (PTP) family. PTPs are involved in regulation of cell growth, differentiation, mitotic cycle, and oncogenic transformation. PTPN6 suppresses cancer cell growth and increases apoptosis [56]. It has also been reported to be downregulated in prostate cancer [57–59]. PTPN11 is known to be overexpressed in breast [60], cervical [61], laryngeal [62] and gastric cancers [63] but down regulated in colon cancer [64].

### Identification of novel molecules involved in curcumin-mediated signaling

In addition to molecules reported in literature to be regulated by curcumin, we identified several kinases which have not been reported previously in curcumin-mediated signaling. One such molecule is pseudopodium-enriched atypical kinase 1 (PEAK1) which is a member of the new kinase family three (NFK3) family. It plays an important role in regulation of cell migration, proliferation and cancer metastasis [65]. We identified a two-fold decrease in phosphorylation of PEAK1 at Y635 upon curcumin treatment. Further, phosphorylation of PEAK1 at Y635 is associated with acinar growth and cell invasion [66]. A member of the TYR family of protein kinase, Fyn-related Src family tyrosine kinase (FRK), also showed a two-fold decrease in phosphorylation at Y497 upon curcumin treatment [Fig. 4a (iii)]. FRK is a non-receptor protein tyrosine-kinase and is known to be involved in migration and invasion. Although there are several reports on the site being phosphorylated, the significance of the phosphorylation site and its role in the function of FRK is currently unknown. Although several sites on kinases were observed to be hypophosphorylated by curcumin, in the case of EPHA2, a member of Eph receptor tyrosine kinase family; we observed a 1.7 fold increase in the phosphorylation levels at Y772. This is further supported by Western blot analysis, which shows hyper-phosphorylation of EPHA2 at Y772 in the presence of curcumin (Fig. 4b).

Protein tyrosine phosphatases are known to regulate a variety of cellular processes including cell growth, differentiation, mitotic cycle, and oncogenic transformation. PTPRK is a protein tyrosine phosphatase (PTP) that is known to regulate a variety of cellular processes including cell growth, differentiation, mitotic cycle, and oncogenic transformation [67, 68]. It negatively regulates STAT3 phosphorylation at Y705 [69]. Upon curcumin exposure, STAT3 phosphorylation decreases at Y705 [65]. In our analysis, phosphorylation of PTPRK at Y858 increased twofold upon curcumin exposure [Fig. 4a (iv)], indicating curcumin regulates STAT3 phosphorylation through PTPRK.

Some of the phosphatases regulated by curcumin and reported for the first time in this study include protein tyrosine phosphatase, receptor type, E (PTPRE) and PTPN6. PTPRE phosphorylation at Y638 is necessary for its activation and regulates the activity of c-SRC. The activity of c-Src is important for maintaining malignant transformation of tumor cells [66]. Our data demonstrates that curcumin can effectively inhibit PTPRE phosphorylation at Y638. Further, c-Src has been reported to phosphorylate GRB2-associated binding protein 1 (GAB1) at Y406 and mediate growth-factor signaling [70]. GAB1 plays a central role in cellular growth response, transformation and apoptosis. Down regulation of GAB1 reduces proliferation and migration in cholangiocarcinoma [71]. Our data shows a two-fold decrease in phosphorylation of GAB1 at Y406 upon curcumin treatment.

### Curcumin induced signaling and apoptosis

Curcumin induces programmed cell death (apoptosis) in many cancer cell types. In our data we identified differential phosphorylation of multiple proteins by curcumin, which have been previously reported in literature to be involved in apoptosis of cancer cells. Caveolin-1 (CAV-1) is a major integral membrane protein on caveolae and its loss of function leads to tumorigenesis. It is known that several drugs such as bromocriptine and taxol increase the phosphorylation of caveolin-1 at Y14 leading to apoptosis in pituitary adenoma and breast cancer [72, 73]. Interestingly, in our analysis we observed a fourfold increased phosphorylation of caveolin-1 at Y14. Further studies are needed to understand the exact mechanism of curcumin-induced phosphorylation of caveolin and its role in apoptosis, which is beyond the scope of this study. Members of the MAPKs family regulate diverse signal transduction pathways that control multiple aspects of cellular physiology, including cell growth, differentiation, and apoptosis [74]. Stress responsive signals have been shown to activate MAPK9, MAPK10 and MAPK12. Drugs such as doxorubicin, apilidin and resveratrol increase phosphorylation of MAPK9 and MAPK12 at Y185 and induce apoptosis [74]. In our data, both MAPK9 and MAPK12 were hyperphosphorylated two and threefold respectively at Y185 in the presence of curcumin.

### Curcumin mediated FAK signaling

**Fig. 5:**
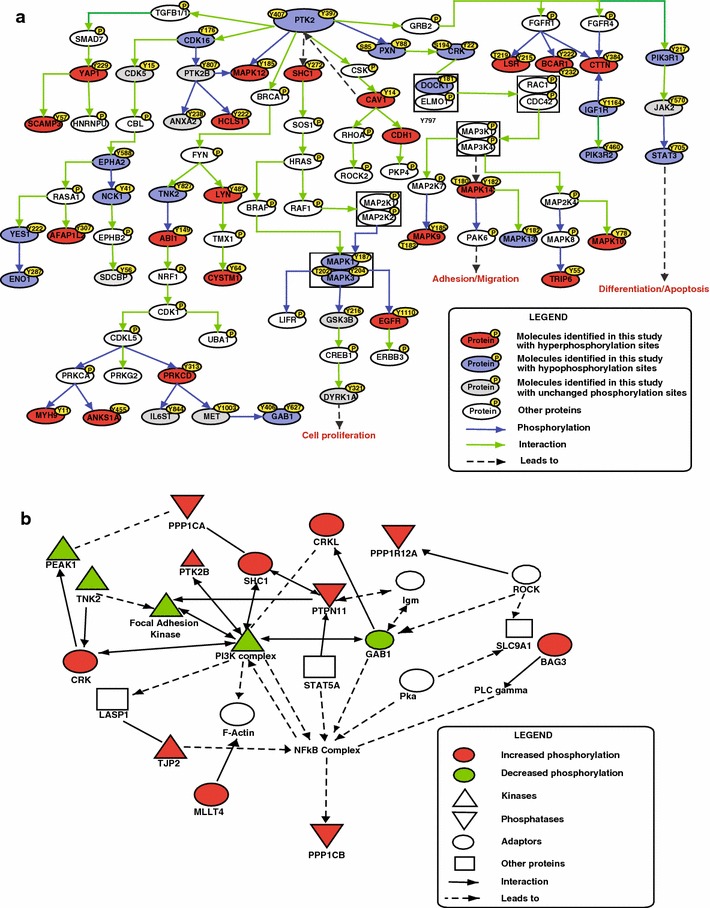
Curcumin regulated signaling networks. **a** Pathway analysis of proteins differentially phosphorylated upon curcumin treatment indicated enrichment of focal adhesion pathway. Proteins identified in this study regulated by curcumin are represented in *red* (hyperphosphorylated) or *green* (hypophosphorylated). **b** Ingenuity network analysis of phosphoproteins regulated by curcumin reveals enrichment of NF-kB sub-network. *Solid lines* represent protein–protein interactions, *dashed lines* with an *arrow* represent enzymatic reactions and *solid lines* with an *arrow* represent both protein-protein interactions and enzymatic reactions

The most significant biological networks identified in IPA analysis (Table 2) which received an IPA score of 37, included several proteins that were differentially expressed in our data and correlated with FAK signaling pathway. FAK is a cytoplasmic tyrosine kinase which influences various signaling pathways that promote cancer growth and metastasis. It controls cell motility, invasion and cell survival [75–77]. Curcumin inhibits phosphorylation of FAK and affects the expression of several extracellular matrix components which play an important role in invasion and metastasis [78]. In agreement with previous studies, we observed curcumin-mediated decreased phosphorylation of FAK at Y397 and Y407. Studies indicate that hyper-activation of FAK through phosphorylation at these sites leads to migration, proliferation and invasion of cells [79–81]. Although inhibition of FAK activation through curcumin is well studied minimal information is available about its downstream signaling. Based on manual literature curation and functional analysis, we identified several proteins, such as CAV1, PI3 K and ERK1 in the focal adhesion pathway (Fig. 5a). Our data shows a four-fold increase in the phosphorylation of CAV1 at Y14 upon curcumin treatment. It is known that over expression of CAV1 reduces the expression of integrin β3 and activity of FAK [82]. Phosphorylation of FAK in response to integrin leads to the formation of phosphotyrosine docking sites for Paxillin which in turn phosphorylates CRK leading to complex formation with DOCK1/ELMO, which ultimately regulates cell migration [83]. FAK also interacts with SHC1 which then recruits SOS1, HRAS, BRAF and activates ERK1/ERK2 kinases that controls cell proliferation [84]. In our analysis, we observed 0.4 and 0.5-fold decreased phosphorylation of the activation sites of ERK1 (T202/Y204)/ERK2 (T185/Y187) respectively, in the curcumin treated cells. Similar results were also observed with the immunoblotting suggesting curcumin mediated inhibition of cellular proliferation.

Curcumin is also known to suppress invasion and migration through inhibition of PI3K/AKT signaling [85]. In agreement with previous studies, our data confirms that curcumin alters the PI3K network. Several studies have shown the role of FAK and PI3K signaling in regulation of integrin signaling [86, 87]. Additionally, network analysis of differentially phosphorylated proteins shows enrichment of PI3 K and NF-κB sub networks for cellular movement, cellular assembly and organization (Fig. 5b). We also observed decreased phosphorylation of GAB1 upon curcumin treatment which contributes to the activation of the PI3K cascade [88].

## Conclusion

Several studies have demonstrated that curcumin has antioxidant, anti-inflammatory, anti-proliferative and pro-apoptotic effects. In this study, we investigated the alterations in tyrosine phosphorylation upon curcumin treatment in cancer cells. We have identified several known curcumin regulated phosphosites, as well as several sites which have not been previously reported. The data obtained in this study will help in better understanding of curcumin-induced signaling in cancer cells. Tyrosine kinases, identified in our study could serve as potential targets for treatment of several cancers. In conclusion, this study represents the first phosphotyrosine proteome analysis of curcumin signaling and provides a rich resource of potential therapeutic targets for cancer.

## Additional files

10.1186/s12014-016-9114-0 (A) Cell viability of CAL 27 with indicated concentrations of curcumin. Classification of differentially phosphorylated proteins based on (B) Localization (C) Biological process (D) Molecular function.
10.1186/s12014-016-9114-0 A list of phosphoPSMs identified by MASCOT and SEQUEST.
10.1186/s12014-016-9114-0 A list of phosphopeptides quantitated by SILAC.

## Abbreviations

HNSCC: head and neck squamous cell carcinoma
IAP: immunoaffinity purification
SILAC: stable isotope labeling by amino acid in cell culture

## References

[1] Rao CV, Rivenson A, Simi B, Reddy BS (1995). Chemoprevention of colon carcinogenesis by dietary curcumin, a naturally occurring plant phenolic compound. Cancer Res.

[2] Farazuddin M, Dua B, Zia Q, Khan AA, Joshi B, Owais M (2014). Chemotherapeutic potential of curcumin-bearing microcells against hepatocellular carcinoma in model animals. Int J Nanomed.

[3] Sreejayan Rao MN (1994). Curcuminoids as potent inhibitors of lipid peroxidation. J Pharm Pharmacol.

[4] Li Q, Chen J, Luo S, Xu J, Huang Q, Liu T (2015). Synthesis and assessment of the antioxidant and antitumor properties of asymmetric curcumin analogues. Eur J Med Chem.

[5] Strimpakos AS, Sharma RA (2008). Curcumin: preventive and therapeutic properties in laboratory studies and clinical trials. Antioxid Redox Signal.

[6] Sahbaie P, Sun Y, Liang DY, Shi XY, Clark JD (2014). Curcumin treatment attenuates pain and enhances functional recovery after incision. Anesth Analg.

[7] Rana C, Piplani H, Vaish V, Nehru B, Sanyal SN (2015). Downregulation of telomerase activity by diclofenac and curcumin is associated with cell cycle arrest and induction of apoptosis in colon cancer. Tumour Biol.

[8] Ramachandran C, Fonseca HB, Jhabvala P, Escalon EA, Melnick SJ (2002). Curcumin inhibits telomerase activity through human telomerase reverse transcritpase in MCF-7 breast cancer cell line. Cancer Lett.

[9] Dorai T, Cao YC, Dorai B, Buttyan R, Katz AE (2001). Therapeutic potential of curcumin in human prostate cancer. III. Curcumin inhibits proliferation, induces apoptosis, and inhibits angiogenesis of LNCaP prostate cancer cells in vivo. Prostate.

[10] Yu J, Zhou X, He X, Dai M, Zhang Q (2011). Curcumin induces apoptosis involving bax/bcl-2 in human hepatoma SMMC-7721 cells. Asian Pac J Cancer Prev.

[11] Cai XZ, Huang WY, Qiao Y, Du SY, Chen Y, Chen D (2013). Inhibitory effects of curcumin on gastric cancer cells: a proteomic study of molecular targets. Phytomedicine.

[12] Wang JB, Qi LL, Zheng SD, Wu TX (2009). Curcumin induces apoptosis through the mitochondria-mediated apoptotic pathway in HT-29 cells. J Zhejiang Univ Sci B.

[13] Gogada R, Amadori M, Zhang H, Jones A, Verone A, Pitarresi J (2011). Curcumin induces Apaf-1-dependent, p21-mediated caspase activation and apoptosis. Cell Cycle.

[14] Shankar S, Chen Q, Sarva K, Siddiqui I, Srivastava RK (2007). Curcumin enhances the apoptosis-inducing potential of TRAIL in prostate cancer cells: molecular mechanisms of apoptosis, migration and angiogenesis. J Mol Signal.

[15] Chen A, Xu J, Johnson AC (2006). Curcumin inhibits human colon cancer cell growth by suppressing gene expression of epidermal growth factor receptor through reducing the activity of the transcription factor Egr-1. Oncogene.

[16] Hong RL, Spohn WH, Hung MC (1999). Curcumin inhibits tyrosine kinase activity of p185neu and also depletes p185neu. Clin Cancer Res.

[17] Camacho-Barquero L, Villegas I, Sanchez-Calvo JM, Talero E, Sanchez-Fidalgo S, Motilva V (2007). Curcumin, a *Curcuma longa* constituent, acts on MAPK p38 pathway modulating COX-2 and iNOS expression in chronic experimental colitis. Int Immunopharmacol.

[18] Rana C, Piplani H, Vaish V, Nehru B, Sanyal SN (2015). Downregulation of PI3-K/Akt/PTEN pathway and activation of mitochondrial intrinsic apoptosis by Diclofenac and Curcumin in colon cancer. Mol Cell Biochem.

[19] Park MJ, Kim EH, Park IC, Lee HC, Woo SH, Lee JY (2002). Curcumin inhibits cell cycle progression of immortalized human umbilical vein endothelial (ECV304) cells by up-regulating cyclin-dependent kinase inhibitor, p21WAF1/CIP1, p27KIP1 and p53. Int J Oncol.

[20] Huang MT, Smart RC, Wong CQ, Conney AH (1988). Inhibitory effect of curcumin, chlorogenic acid, caffeic acid, and ferulic acid on tumor promotion in mouse skin by 12-*O*-tetradecanoylphorbol-13-acetate. Cancer Res.

[21] Lin YC, Chen HW, Kuo YC, Chang YF, Lee YJ, Hwang JJ (2010). Therapeutic efficacy evaluation of curcumin on human oral squamous cell carcinoma xenograft using multimodalities of molecular imaging. Am J Chin Med.

[22] Yu S, Shen G, Khor TO, Kim JH, Kong AN (2008). Curcumin inhibits Akt/mammalian target of rapamycin signaling through protein phosphatase-dependent mechanism. Mol Cancer Ther.

[23] Zhen L, Fan D, Yi X, Cao X, Chen D, Wang L (2014). Curcumin inhibits oral squamous cell carcinoma proliferation and invasion via EGFR signaling pathways. Int J Clin Exp Pathol.

[24] Wu J, Patmore DM, Jousma E, Eaves DW, Breving K, Patel AV (2014). EGFR-STAT3 signaling promotes formation of malignant peripheral nerve sheath tumors. Oncogene.

[25] Gaedeke J, Noble NA, Border WA (2004). Curcumin blocks multiple sites of the TGF-beta signaling cascade in renal cells. Kidney Int.

[26] Guo Y, Shan Q, Gong Y, Lin J, Shi F, Shi R (2014). Curcumin induces apoptosis via simultaneously targeting AKT/mTOR and RAF/MEK/ERK survival signaling pathways in human leukemia THP-1 cells. Pharmazie.

[27] Siwak DR, Shishodia S, Aggarwal BB, Kurzrock R (2005). Curcumin-induced antiproliferative and proapoptotic effects in melanoma cells are associated with suppression of IkappaB kinase and nuclear factor kappaB activity and are independent of the B-Raf/mitogen-activated/extracellular signal-regulated protein kinase pathway and the Akt pathway. Cancer.

[28] Chen YR, Tan TH (1998). Inhibition of the c-Jun N-terminal kinase (JNK) signaling pathway by curcumin. Oncogene.

[29] Chung SS, Vadgama JV (2015). Curcumin and epigallocatechin gallate inhibit the cancer stem cell phenotype via down-regulation of STAT3-NFkappaB signaling. Anticancer Res.

[30] Hunter T (2009). Tyrosine phosphorylation: thirty years and counting. Curr Opin Cell Biol.

[31] D’Aguanno S, D’Agnano I, De Canio M, Rossi C, Bernardini S, Federici G (2012). Shotgun proteomics and network analysis of neuroblastoma cell lines treated with curcumin. Mol BioSyst.

[32] Fang HY, Chen SB, Guo DJ, Pan SY, Yu ZL (2011). Proteomic identification of differentially expressed proteins in curcumin-treated MCF-7 cells. Phytomedicine.

[33] Madden K, Flowers L, Salani R, Horowitz I, Logan S, Kowalski K (2009). Proteomics-based approach to elucidate the mechanism of antitumor effect of curcumin in cervical cancer. Prostaglandins Leukot Essent Fatty Acids.

[34] Liao S, Xia J, Chen Z, Zhang S, Ahmad A, Miele L (2011). Inhibitory effect of curcumin on oral carcinoma CAL-27 cells via suppression of Notch-1 and NF-kappaB signaling pathways. J Cell Biochem.

[35] LoTempio MM, Veena MS, Steele HL, Ramamurthy B, Ramalingam TS, Cohen AN (2005). Curcumin suppresses growth of head and neck squamous cell carcinoma. Clin Cancer Res.

[36] Rappsilber J, Ishihama Y, Mann M (2003). Stop and go extraction tips for matrix-assisted laser desorption/ionization, nanoelectrospray, and LC/MS sample pretreatment in proteomics. Anal Chem.

[37] Pinto SM, Nirujogi RS, Rojas PL, Patil AH, Manda SS, Subbannayya Y (2015). Quantitative phosphoproteomic analysis of IL-33-mediated signaling. Proteomics.

[38] Prasad TS, Kandasamy K, Pandey A (2009). Human Protein Reference Database and Human Proteinpedia as discovery tools for systems biology. Methods Mol Biol.

[39] da Huang W, Sherman BT, Lempicki RA (2009). Systematic and integrative analysis of large gene lists using DAVID bioinformatics resources. Nat Protoc.

[40] Chou MF, Schwartz D (2011). Biological sequence motif discovery using motif-x. Curr Protoc Bioinformatics.

[41] Nanjappa V, Renuse S, Sathe GJ, Raja R, Syed N, Radhakrishnan A (2015). Chronic exposure to chewing tobacco selects for overexpression of stearoyl-CoA desaturase in normal oral keratinocytes. Cancer Biol Ther.

[42] Subbannayya Y, Syed N, Barbhuiya MA, Raja R, Marimuthu A, Sahasrabuddhe N (2015). Calcium calmodulin dependent kinase kinase 2—a novel therapeutic target for gastric adenocarcinoma. Cancer Biol Ther.

[43] Goel R, Muthusamy B, Pandey A, Prasad TS (2011). Human protein reference database and human proteinpedia as discovery resources for molecular biotechnology. Mol Biotechnol.

[44] Wang JY (2014). The capable ABL: what is its biological function?. Mol Cell Biol.

[45] Roskoski R (2015). Src protein-tyrosine kinase structure, mechanism, and small molecule inhibitors. Pharmacol Res.

[46] Ito T, Ito M, Naito S, Ohtsuru A, Nagayama Y, Kanematsu T (1999). Expression of the Axl receptor tyrosine kinase in human thyroid carcinoma. Thyroid.

[47] Gustafsson A, Martuszewska D, Johansson M, Ekman C, Hafizi S, Ljungberg B (2009). Differential expression of Axl and Gas6 in renal cell carcinoma reflecting tumor advancement and survival. Clin Cancer Res.

[48] Hector A, Montgomery EA, Karikari C, Canto M, Dunbar KB, Wang JS (2010). The Axl receptor tyrosine kinase is an adverse prognostic factor and a therapeutic target in esophageal adenocarcinoma. Cancer Biol Ther.

[49] Lee CH, Yen CY, Liu SY, Chen CK, Chiang CF, Shiah SG (2012). Axl is a prognostic marker in oral squamous cell carcinoma. Ann Surg Oncol.

[50] Gjerdrum C, Tiron C, Hoiby T, Stefansson I, Haugen H, Sandal T (2010). Axl is an essential epithelial-to-mesenchymal transition-induced regulator of breast cancer metastasis and patient survival. Proc Natl Acad Sci USA.

[51] Fang WB, Brantley-Sieders DM, Hwang Y, Ham AJ, Chen J (2008). Identification and functional analysis of phosphorylated tyrosine residues within EphA2 receptor tyrosine kinase. J Biol Chem.

[52] Yoon S, Seger R (2006). The extracellular signal-regulated kinase: multiple substrates regulate diverse cellular functions. Growth Factors.

[53] McCubrey JA, Steelman LS, Chappell WH, Abrams SL, Wong EW, Chang F (2007). Roles of the Raf/MEK/ERK pathway in cell growth, malignant transformation and drug resistance. Biochim Biophys Acta.

[54] Xie YQ, Wu XB, Tang SQ (2014). Curcumin treatment alters ERK-1/2 signaling in vitro and inhibits nasopharyngeal carcinoma proliferation in mouse xenografts. Int J Clin Exp Med.

[55] Liu CW, Wang RH, Berndt N (2006). Protein phosphatase 1alpha activity prevents oncogenic transformation. Mol Carcinog.

[56] Kang SH, Jeong SJ, Kim SH, Kim JH, Jung JH, Koh W (2012). Icariside II induces apoptosis in U937 acute myeloid leukemia cells: role of inactivation of STAT3-related signaling. PLoS One.

[57] Wu C, Guan Q, Wang Y, Zhao ZJ, Zhou GW (2003). SHP-1 suppresses cancer cell growth by promoting degradation of JAK kinases. J Cell Biochem.

[58] Zapata PD, Ropero RM, Valencia AM, Buscail L, Lopez JI, Martin-Orozco RM (2002). Autocrine regulation of human prostate carcinoma cell proliferation by somatostatin through the modulation of the SH2 domain containing protein tyrosine phosphatase (SHP)-1. J Clin Endocrinol Metab.

[59] Tassidis H, Culig Z, Wingren AG, Harkonen P (2010). Role of the protein tyrosine phosphatase SHP-1 in Interleukin-6 regulation of prostate cancer cells. Prostate.

[60] Hu Z, Fang H, Wang X, Chen D, Chen Z, Wang S (2014). Overexpression of SHP2 tyrosine phosphatase promotes the tumorigenesis of breast carcinoma. Oncol Rep.

[61] Meng F, Zhao X, Zhang S (2012). Expression and significance of SHP-2 in human papillomavirus infected cervical cancer. J Huazhong Univ Sci Technolog Med Sci.

[62] Gu J, Han T, Ma RH, Zhu YL, Jia YN, Du JJ (2014). SHP2 promotes laryngeal cancer growth through the Ras/Raf/Mek/Erk pathway and serves as a prognostic indicator for laryngeal cancer. Int J Oncol.

[63] Dong S, Li FQ, Zhang Q, Lv KZ, Yang HL, Gao Y (2012). Expression and clinical significance of SHP2 in gastric cancer. J Int Med Res.

[64] Cai P, Guo W, Yuan H, Li Q, Wang W, Sun Y (2014). Expression and clinical significance of tyrosine phosphatase SHP-2 in colon cancer. Biomed Pharmacother.

[65] Wang Y, Kelber JA, Tran Cao HS, Cantin GT, Lin R, Wang W (2010). Pseudopodium-enriched atypical kinase 1 regulates the cytoskeleton and cancer progression [corrected]. Proc Natl Acad Sci USA.

[66] Croucher DR, Hochgrafe F, Zhang L, Liu L, Lyons RJ, Rickwood D (2013). Involvement of Lyn and the atypical kinase SgK269/PEAK1 in a basal breast cancer signaling pathway. Cancer Res.

[67] Sun PH, Ye L, Mason MD, Jiang WG (2013). Protein tyrosine phosphatase kappa (PTPRK) is a negative regulator of adhesion and invasion of breast cancer cells, and associates with poor prognosis of breast cancer. J Cancer Res Clin Oncol.

[68] Erdenebayar N, Maekawa Y, Nishida J, Kitamura A, Yasutomo K (2009). Protein-tyrosine phosphatase-kappa regulates CD4+ T cell development through ERK1/2-mediated signaling. Biochem Biophys Res Commun.

[69] Chen YW, Guo T, Shen L, Wong KY, Tao Q, Choi WW (2015). Receptor-type tyrosine-protein phosphatase kappa directly targets STAT3 activation for tumor suppression in nasal NK/T-cell lymphoma. Blood.

[70] Chan PC, Sudhakar JN, Lai CC, Chen HC (2010). Differential phosphorylation of the docking protein Gab1 by c-Src and the hepatocyte growth factor receptor regulates different aspects of cell functions. Oncogene.

[71] Sang H, Li T, Li H, Liu J (2013). Down-regulation of Gab1 inhibits cell proliferation and migration in hilar cholangiocarcinoma. PLoS One.

[72] Jiang YN, Li YH, Ke MW, Tseng TY, Tang YB, Huang MC (2007). Caveolin-1 sensitizes rat pituitary adenoma GH3 cells to bromocriptine induced apoptosis. Cancer cell Int.

[73] Shajahan AN, Wang A, Decker M, Minshall RD, Liu MC, Clarke R (2007). Caveolin-1 tyrosine phosphorylation enhances paclitaxel-mediated cytotoxicity. J Biol Chem.

[74] Kim EK, Choi EJ (2010). Pathological roles of MAPK signaling pathways in human diseases. Biochim Biophys Acta.

[75] Shibue T, Brooks MW, Inan MF, Reinhardt F, Weinberg RA (2012). The outgrowth of micrometastases is enabled by the formation of filopodium-like protrusions. Cancer Discov.

[76] Cance WG, Golubovskaya VM (2008). Focal adhesion kinase versus p53: apoptosis or survival?. Science signaling..

[77] Mitra SK, Hanson DA, Schlaepfer DD (2005). Focal adhesion kinase: in command and control of cell motility. Nat Rev Mol Cell Biol.

[78] Chen CC, Sureshbabul M, Chen HW, Lin YS, Lee JY, Hong QS (2013). Curcumin suppresses metastasis via Sp-1, FAK inhibition, and E-cadherin upregulation in colorectal cancer. Evid Based Complement Alternat Med.

[79] Quizi JL, Baron K, Al-Zahrani KN, O’Reilly P, Sriram RK, Conway J (2013). SLK-mediated phosphorylation of paxillin is required for focal adhesion turnover and cell migration. Oncogene.

[80] Parri M, Buricchi F, Giannoni E, Grimaldi G, Mello T, Raugei G (2007). EphrinA1 activates a Src/focal adhesion kinase-mediated motility response leading to rho-dependent actino/myosin contractility. J Biol Chem.

[81] Mukhopadhyay NK, Gordon GJ, Chen CJ, Bueno R, Sugarbaker DJ, Jaklitsch MT (2005). Activation of focal adhesion kinase in human lung cancer cells involves multiple and potentially parallel signaling events. J Cell Mol Med.

[82] Trimmer C, Whitaker-Menezes D, Bonuccelli G, Milliman JN, Daumer KM, Aplin AE (2010). CAV1 inhibits metastatic potential in melanomas through suppression of the integrin/Src/FAK signaling pathway. Cancer Res.

[83] Kiyokawa E, Hashimoto Y, Kurata T, Sugimura H, Matsuda M (1998). Evidence that DOCK180 up-regulates signals from the CrkII-p130(Cas) complex. J Biol Chem.

[84] Schlaepfer DD, Hunter T (1997). Focal adhesion kinase overexpression enhances ras-dependent integrin signaling to ERK2/mitogen-activated protein kinase through interactions with and activation of c-Src. J Biol Chem.

[85] Xu X, Qin J, Liu W (2014). Curcumin inhibits the invasion of thyroid cancer cells via down-regulation of PI3K/Akt signaling pathway. Gene.

[86] Wu J, Li Y, Dang YZ, Gao HX, Jiang JL, Chen ZN (2014). HAb18G/CD147 promotes radioresistance in hepatocellular carcinoma cells: a potential role for integrin beta1 signaling. Mol Cancer Ther.

[87] Niu G, Ye T, Qin L, Bourbon PM, Chang C, Zhao S (2015). Orphan nuclear receptor TR3/Nur77 improves wound healing by upregulating the expression of integrin beta4. FASEB J.

[88] Eulenfeld R, Schaper F (2009). A new mechanism for the regulation of Gab1 recruitment to the plasma membrane. J Cell Sci.

